# If there is smoke, there must be fire – Isolated distal, non-displaced, intraarticular ulna fracture: A case report

**DOI:** 10.1016/j.ijscr.2019.06.009

**Published:** 2019-06-12

**Authors:** Marie Hélène Manz, Kai Oliver Jensen, Florin Allemann, Hans-Peter Simmen, Thomas Rauer

**Affiliations:** University Hospital Zurich, Division of Trauma Surgery, Rämistrasse 100, 8091 Zurich, Switzerland

**Keywords:** Distal ulna fracture, Forearm, Wrist, Isolated fracture, Trauma

## Abstract

**Introduction:**

Falling on the outstretched arm is a frequent trauma. The consequences can be isolated fractures of the distal radius, combined fractures of the distal radius and distal ulna - whereas the isolated fracture of the distal ulna is an uncommon trauma.

**Presentation of case:**

We report on a 20- year- old male who presented at the emergency department after a motorcycle accident, suffering pain at his left forearm and left wrist. After an unremarkable x-ray in two planes, a CT- scan of the left wrist was performed and detected an isolated distal ulna fracture. The patient was treated with a forearm cast which led to a satisfying long term outcome.

**Discussion:**

Based on the presented case the anatomical peculiarities of the distal radioulnar joint and the different trauma mechanisms as well as the resulting lesions, the diagnostic modalities and the treatment options are discussed.

**Conclusion:**

We recommend performing an additional CT scan of the wrist in case of credible complaints after trauma to avoid baulking this very rare type of fracture.

## Highlights

• Isolated distal ulna styloid fractures are a rarity.
• Timely diagnosis and appropriate therapy reduce posttraumatic morbidity.
• High level of suspicion should be maintained in the presence of nonspecific pain, even if the initial X-ray is inconspicuous.

## 1 Introduction

The present work has been reported in line with the SCARE criteria [1].

With a majority of 25 percent, the fracture of the distal radius is the most common type of fracture. In older people, their appearance is associated with minor trauma, like fall from the feet. Depending on the accident a fall on the outstretched forearm can result in Smith’ fracture or in Colles’ fracture. Even direct trauma is possible [2,3]. Fractures of the distal ulna are rare. They tend to occur in association with fractures to the distal radius in 5–10 %, disregarding fractures of the ulnar styloid [2,4]. Isolated fractures are usually caused by direct trauma [2,3]. They are even more seldom and their significance is often underestimated [2,3]. The ulna is a long tubular bone interacting with the radius in two joints. Between the elbow and the distal radioulnar joint (DRUJ) they form a functional unit. Injuries to the DRUJ as fractures with intraarticular gaps, shortening or angulation of the ulna can destroy the integrity of the stable construct and the functional unit of the joint and finally result in a lack of mobility [2,4].

The diagnosis of a fracture of the wrist is determined of medical history, clinical examination and x-ray in two plains [2,3]. In cases, such as the following, with a mismatch between clinical suspicion and standard-radiological finding, the enforcement of a diagnosis availing extended diagnostic investigations is necessary.

## 2 Case report

We report on a 20- year-old healthy male, who presented at the surgical emergency department complaining about pain at the left forearm and wrist ensuing a fall from his motorcycle without third party interference. The patient reported of an initial driving speed of 10–20 mph. Helmet and protective clothing was worn.

**Fig. 1:**
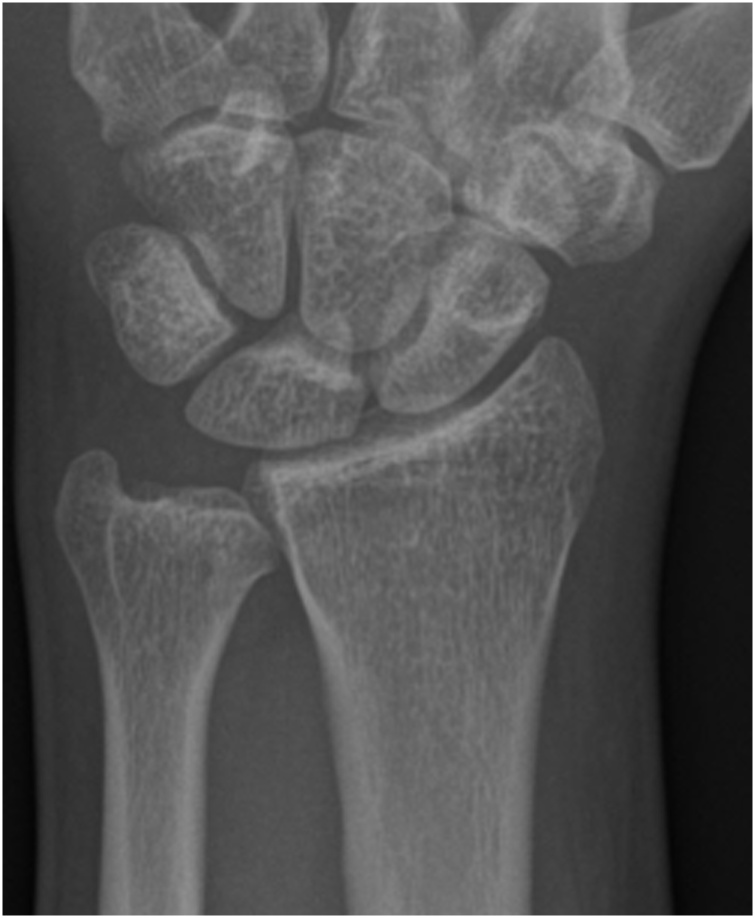
Initial AP radiography of the patient`s left wrist.

Due to the anamnestic information and clinical examination an x-ray of the left hand and forearm was performed. It showed no pathologic results (Fig. 1).

**Fig. 2:**
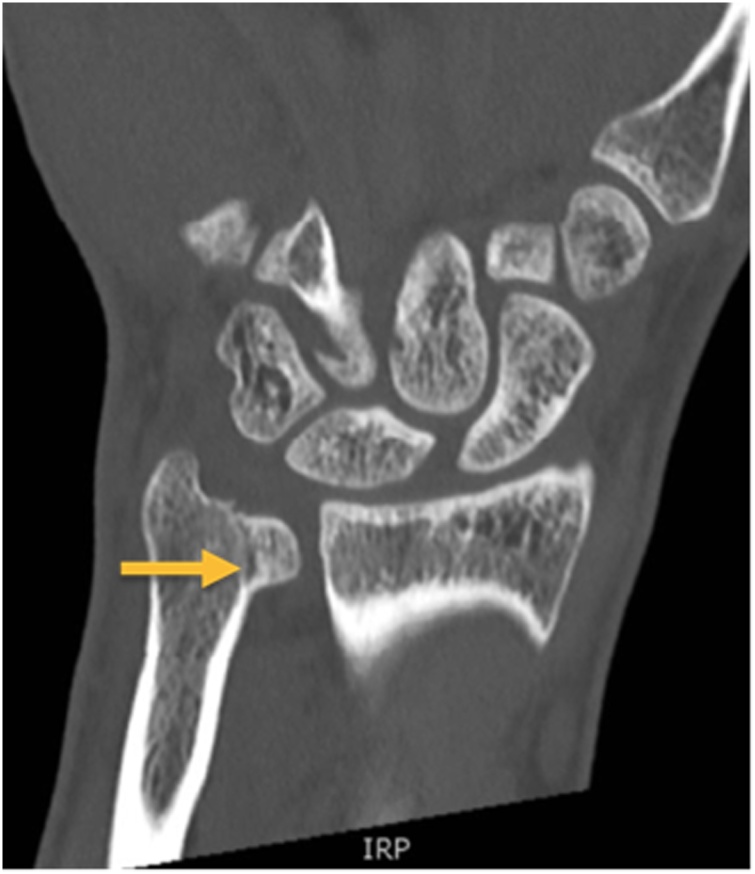
CT scan of the patient`s left wrist (coronal view) showing an undisplaced Fracture of the ulna styloid (arrow).

**Fig. 3:**
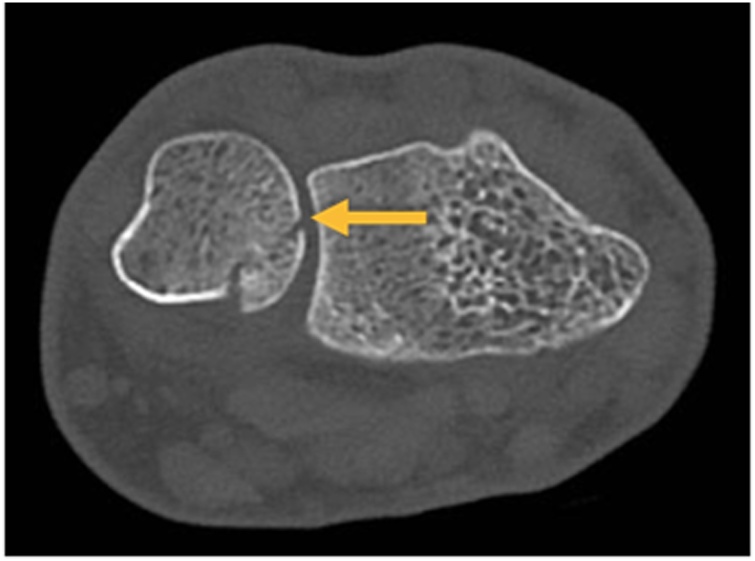
CT scan of the patient`s left wrist (axial view) showing an undisplaced Fracture of the ulna styloid (arrow).

With respect to the clinical suspicion of an osseous injury to the left wrist, positive fovea ulnaris sign and Triangular Fibro- Cartilage Complex (TFCC) load test, an additional computed-tomography was initiated. It revealed the diagnosis of a distal, non-displaced, intraarticular ulna fracture with involvement of the ulnocarpal and the radioulnar joint (Fig. 2, Fig. 3).

The patient got an outpatient treatment immobilized by an intrinsic plus forearm cast and an appointment for a clinical 1- week follow-up was scheduled.

By this point, local examination showed a moderate swelling of the wrist. He described slight pain of the ulnar wrist and forearm. Due to this, the pronation and ulnar deviation was compromised. The patient was treated with a closed forearm brace for another 4 weeks under avoidance of supination and pronation.

He was seen again 5 weeks after trauma denying any pain. Neither swelling, nor instability of the DRUJ was eminent. Painless range of motion with solely terminal limitations (pro-/ supination 85/0/85°, dorsal extension/ palmar flexion 35/0/50°, radial abduction/ ulnar abduction 25/0/30°). A performed x-ray of the left wrist showed no secondary dislocation. The patient was discharged with the proviso of full weight bearing and free range of motion.

**Fig. 4:**
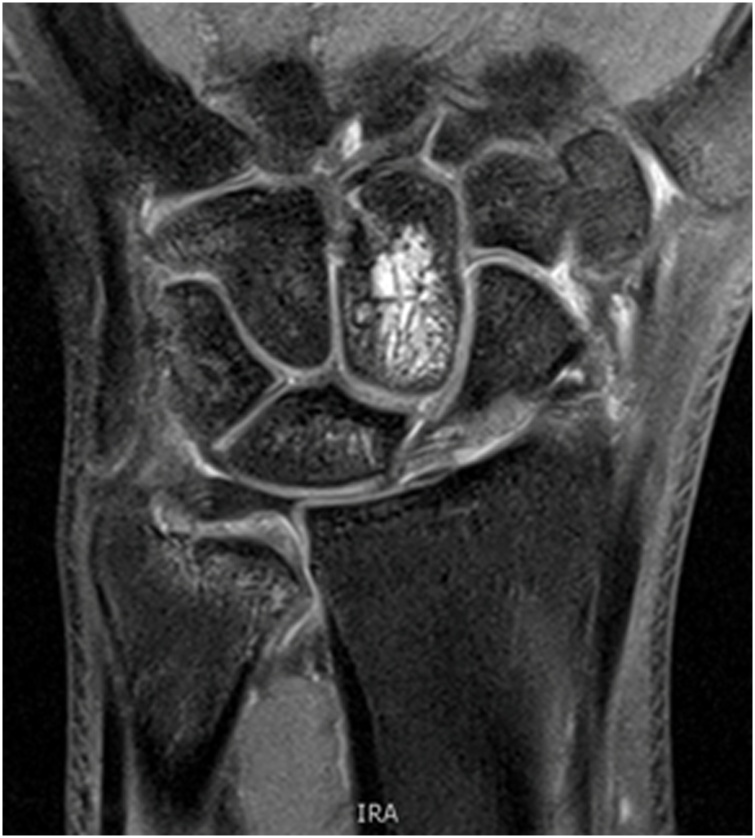
MRI of the patient`s wrist (coronal view).

Following a planned outpatient MRI of the wrist a 10- week follow- up of the patient showed no significant changes of the results. The MRI revealed the integrity of the TFCC and an osseous consolidation of the fracture with a persistent bone marrow edema (Fig. 4). No further follow-up has been scheduled.

## 3 Discussion

This case illustrates the important role of suitable additional imaging in case of clinical presumption of a fracture that can remain undetected for the examiner in the conventional x-ray.

Usually fractures of the distal ulna occur as concomitant injuries [2]. As described in literature by Richards et al. and Logan et al. relating to a surgical treatment various methods exist, such as Kirschner wires, tension banding, intraosseous wiring or plate/screw constructs [2,3]. Fractures of the ulnar head with an intraarticular step or instability are treated with Kirschner wires or locking plates [2]. To achieve postoperative free range of motion without restrictions, therapy of every fracture consists in restoration of the anatomical articular surface, adjustment and fixation of differences in length, axis and rotation of both bones of the forearm [5]. These days, fractures of the distal forearm are rarely treated conservatively [5].

The decision on a conservative treatment of the fracture of our patient initially was made based on the integrity of the ulnar styloid and the non- displacement of the fracture in the medical imaging moreover in the computed tomography, which supported us to accurately define the fracture fragments [4].

Major stability of the wrist is performed by the DRUJ. Consisting of ligaments it builds a functional unit between the distal ulna and radius [2] and represents the peripheral part of the TFCC [2,4,5]. Regarding the stability of the DRUJ, the ulnar head plays a central role [4]. Fractures in this area can result in a dysfunction of the joint by variations in joint axis of rotation. The fovea ulnaris builds the center axis of ulnar head rotation and builds the attachment for the deep components of the DRUJ and the TFCC [4]. Additional, as described by Palmer et al. the ulna carries about 20% of the axial load of the forearm through its articulation with the medial carpus along with the TFCC [6]. Therefore, injuries of the TFCC or fractures of the bony suspensory and related changes in the functional unit of the forearm and wrist can cause severe physiological alterations [6]. Our patient had negative initial standard radiographs but showed persistence of pain of the distal ulna. He had pain on palpation of the ulna and a positive fovea ulnaris sign and TFCC load test. To preclude an eventual bony injury we made a decision to perform the additional CT scan. As described by Richards et al. it is unclear what the associations are between isolated distal ulna fractures and TFCC injuries. But if a TFCC injury or disruption of the DRUJ is suspected, MRI may be considered [2]. Due to the suspicious clinical examination of the wrist and the existing possibility of an injury of the TFCC complex, we decided to perform an additional MRI in case of persistence of symptoms and pains.

In the end, the MRI of the wrist showed residual structured bonebruise on the metacarpals. A ligament injury could be excluded.

## 4 Conclusion

The presented case emphasizes the importance of the initial clinical assessment and the additional radiological imaging in the presence of nonspecific pain even if the initial X-ray is inconspicuous.

## Declaration of Competing Interest

No conflict of interest.

## Sources of funding

No source of funding.

## Ethical approval

Not applicable.

As this submission is a single case report an ethical approval is not necessary. But written informed consents was obtained from the patient for publication of this case report and any accompanying images.

## Consent

Written informed consents was obtained from the patient for publication of this case report and any accompanying images.

## Author contribution

Marie Hélène Manz: wrote the manuscript.

Kai Oliver Jensen: reviewed the manuscript.

Florin Allemann: reviewed the manuscript.

Hans-Peter Simmen: reviewed the manuscript.

Thomas Rauer: cared for the patient; reviewed the manuscript.

## Registration of research studies

Not necessary.

## Guarantor

Dr. med. Thomas Rauer.

## Provenance and peer review

Not commissioned, externally peer-reviewed.
